# Factors Influencing Functional Outcomes in Supracondylar Humerus Fractures: A Retrospective Study of Paediatric Patients in a Level One Trauma Centre

**DOI:** 10.7759/cureus.37447

**Published:** 2023-04-11

**Authors:** Panagiotis Poulios, Athanasios Serlis, Matthieu Durand-Hill, Georgios Konstantopoulos

**Affiliations:** 1 Orthopaedics and Trauma, Chelsea and Westminster Hospital, London, GBR; 2 Orthopaedics, Peterborough City Hospital, London, GBR; 3 Trauma and Orthopaedics, London North West University Healthcare NHS Trust, London, GBR; 4 Orthopaedics, West Suffolk Hospital, London, GBR

**Keywords:** paediatric orthopaedics, complications, clinician-measured outcomes, patient outcomes, supracondylar humeral fracture

## Abstract

Background

The outcomes after fixation of the supracondylar humerus fracture (SCHF) are not documented in the current literature. In our study, we endeavour to determine the factors that influence the functional outcome and gauge their respective impact.

Methodology

We retrospectively reviewed the outcomes of patients who presented to our tertiary care centre (Royal London Hospital) with SCHFs between September 2017 and February 2018. We analysed patient records to assess several clinical parameters, including age, Gartland’s classification, comorbidities, time to treatment, and fixation configuration. We conducted a multiple linear regression analysis to determine each of the clinical parameter’s impact on the functional and cosmetic outcome, as reflected in Flynn’s criteria.

Results

We included 112 patients in our study. Pediatric SCHFs had good functional outcomes based on Flynn’s criteria. There was no significant statistical difference in functional outcomes with respect to sex (p= 0.713), age (p= 0.96), fracture type (p= 0.14), K-wire configuration (p=0.83), and time elapsed since surgery (p= 0.240).

Conclusions

Our results demonstrate that good functional outcomes can be expected with paediatric SCHFs based on Flynn’s criteria, regardless of age at injury, sex, or pin configuration, provided satisfactory reduction is achieved and maintained. The only variable with statistical significance was Gartland’s grade; Grades III and IV were correlated with poorer outcomes.

## Introduction

Supracondylar fractures of the humerus (SCHF) are a common type of injury in childhood, with the highest prevalence among children aged 3-5 [1,2]. Despite the abundance of current literature on the topic, significant controversy still needs to be addressed. There is a lack of consensus regarding the factors that affect the functional outcome, a clinically accurate and relevant measure [3-5].

Several authors strongly advocate that prognostic factors can be stratified into three categories, namely, patient-related (age, comorbidities, and body mass index (BMI)), injury-related (pattern, severity, and concomitant complications), and surgeon-related (technique, quality of reduction, and time to surgery) [6-8]. However, the question persists regarding how each factor impacts the prognosis and subsequent functional outcome.

The functional outcome and patient satisfaction are strongly linked with restoring the elbow arch of motion in the sagittal plane (flexion and extension). Flynn’s criteria are a well-established outcome measure for supracondylar fractures, assessing the functional and cosmetic scores based on the elbow carrying angle and range of motion (ROM) [9].

This paper describes the experience of a level-one paediatric trauma centre in managing these fractures. Our primary objective is determining the correlation between fracture pattern and wire configuration with functional outcome. Our secondary goal is to evaluate the impact of age, time to treatment, and the presence of neurovascular injury.

## Materials and methods

For six months (from September 2017 to February 2018), we conducted a retrospective analysis of the medical records and radiographs of 184 cases involving supracondylar fractures treated surgically using closed reduction and percutaneous pinning. We excluded patients with multiple open and bilateral injuries, neurovascular complications, head injuries, history of previous pathologies, unclear documentation, or incomplete radiographs from the study. After applying these exclusion criteria, 112 patients remained for analysis. Each patient’s sex and age during the injury were duly documented.

**Figure 1:**
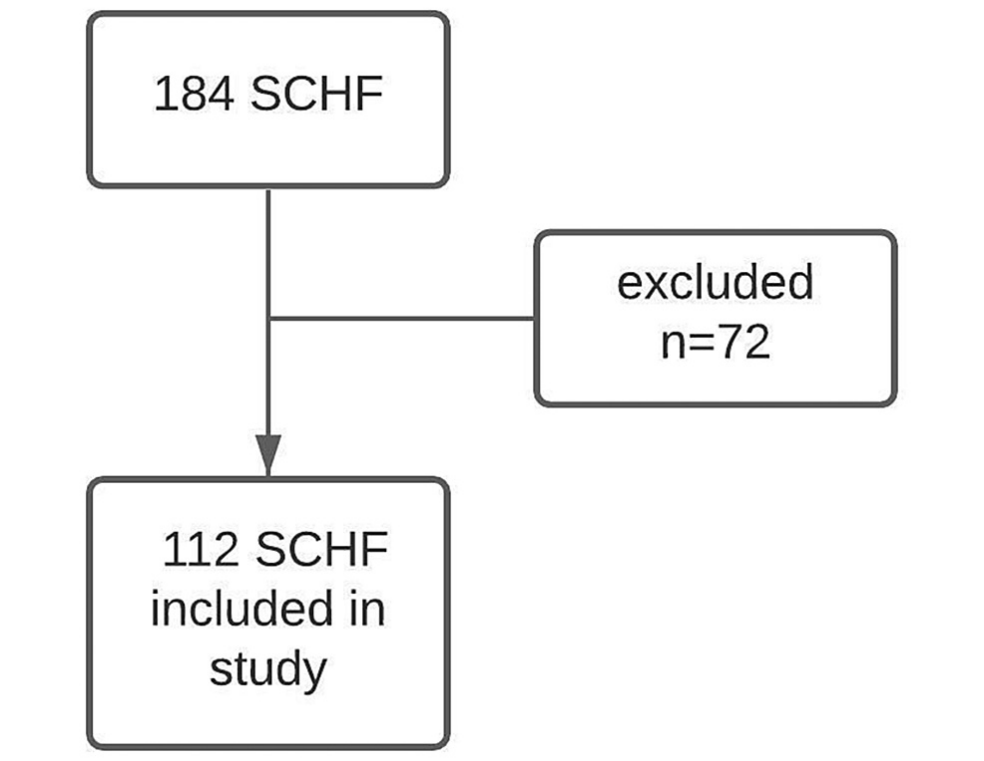
Enrolment of eligible patients. SCHF: supracondylar humerus fracture

We employed Gartland’s classification to ascertain the type and pattern of fracture, which has demonstrated high intra and interobserver concordance and is widely accepted for its reliability [10,11]. Furthermore, we included Grade IV fractures displaced entirely and unstable in both extension and flexion when evaluated intraoperatively. Figure 1 summarises the flowchart of patient enrolment.

Despite the use of Gartland’s classification, the decision for operative management was primarily based on the degree of displacement in the coronal plane, which involved alterations of the Baumann angle and the sagittal plane, which was determined by the anterior humeral line. Specifically, we opted for surgical management if the anterior humeral shaft line did not intersect the capitellum in the lateral views and the posterior periosteal hinge was not intact. Additionally, in the coronal plane, angulation or collapse indicated operative management. Furthermore, the neurovascular compromise was considered an independent criterion for surgical intervention.

Under appropriate marking and with patient consent, the reduction and pinning of the fracture were performed under fluoroscopy, using either a cross or lateral divergent wire configuration, based on the surgeon’s training and experience. Notably, before pinning the medial side, a mini-open incision was made to provide visualisation and protect the ulnar nerve during the procedure, thus avoiding iatrogenic complications. Intraoperatively, the fracture was manipulated to achieve reduction and was further classified as a subset of Gartland’s Grade IV if it was grossly unstable in both extension and flexion. Postoperatively, the limb was immobilised in an above-elbow backslab at 80 degrees of flexion, with the aim of clinical and radiographic review and conversion of the splint into a full cast within seven days after the patient’s discharge.

The next scheduled follow-up appointment was in three weeks, during which the cast and K-wires were removed, and the wound was reviewed along with radiographs. The final review was at 24 weeks when the patient was typically discharged with instructions for mobilisation and self-physiotherapy. During these visits, we assessed the elbow’s carrying angle and arc of motion. Instead of using traditional physical tools, we utilised a digital application called the Goniometer Pro, which can be downloaded from the Apple Store. This application utilises the built-in accelerometer of mobile devices to provide quick and accurate measurements, regardless of the user’s experience level. The functional and cosmetic outcome was tabulated using Flynn’s criteria (Table 1).

**Table 1:** Flynn’s criteria.

Results	Rating	Carrying angle	Loss of motion
	Excellent	0°–5°	0°–5°
Satisfactory	Good	5°–10°	5°–10°
	Fair	10°–15°	10°–15°
Unsatisfactory	Poor	>15°	>15°

Statistical analysis

The data analysis was performed using SPSS version 21 (IBM Corp., Armonk, New York, USA). The chi-squared test was used to compare categorical variables; if expected counts were <5, we instead used Fisher’s exact test.

## Results

Of the 184 patients with SCHF initially considered for our study, 64 were excluded as they did not meet the inclusion criteria. Additionally, eight patients were lost to follow-up due to relocating or opting for follow-up at a different hospital, leaving us with 112 patients for analysis.

Patient demographics

A total of 112 patients, comprising 51 males and 61 females, were included in our study. The mean age of our sample was 68.4 months, with a standard deviation of 63. The mean length of follow-up was 54 days, with a standard deviation of 21. The minimum follow-up duration was 14 days. We observed a higher incidence of supracondylar fractures in girls (68.32%) compared to boys. Additionally, the left elbow was more commonly involved, accounting for 64.96% of cases. Among the fractures, the vast majority (93.28%) were of the extension type, while only a small minority (6.72%) were of the flexion type.

We subdivided the patients according to their fracture type: 36 (32.14%) patients had type II fractures, 65 (58.03%) patients had type III fractures, and nine (8%) patients had type IV fractures. The mean age of patients with type II supracondylar fractures according to Gartland’s classification was 62.97 months (or 5.24 years), 62.86 months (or 5.23 years) for type III fractures, and 60.67 months (or 5.05 years) for type IV fractures. Table 2 provides an overview of the clinical features of the participants, and Table 3 summarises the clinical variables.

**Table 2:** Clinical characteristics of the study participants. SCHF: supracondylar humerus fracture

Clinical characteristic	All patients (n = 112)
Age, mean ± SD	68.4 months
Female	68.32%
Male	31.68%
Type of SCHF (%)	
Extension type	93.28%
Flexion type	6.72%
Gartland fracture grade (%)	
Type II	32.14%
Type III	58.03%
Type IV	8%
Age of the patient with SCHF	
Type II	62.97 months (=5.24 years)
Type III	62.86 months (=5.23 years)
Type IV	60.67 months (=5.05 years).
Vascular impairment	7.14%

**Table 3:** Summary of the clinical variables.

Variable	Flynn’s criteria			
	Excellent (loss <5°), n = 68	Good (loss = 6°–10°), n =25	Fair (loss = 11°–15°), n=11	Poor (Loss >15°), n=8
Sex				
Male (n = 51)	33	11	5	2
Female (n = 61)	35	14	6	6
Gartland’s class				
2 (n = 36)	20	10	6	0
3 (n = 65)	39	14	4	8
4 (n = 10)	9	0	1	0
Pin construct				
Divergent pins (n = 39)	27	5	6	1
Crossed pins (n = 73)	41	20	5	7
Time to treatment				
<24 hours (n = 100)	62	22	8	8
>24 hours (n = 12)	6	3	3	0

Indication for urgent surgery

Emergency surgery was indicated for cases of vascular impairment, characterised by diminished or absent radial pulse and abnormal perfusion. Interestingly, only a paltry proportion (7.14%) of the total cohort (n = 8) fell into this category, and none of them necessitated open exploration. In our series, eight patients exhibited a pre-reduction absence of a radial pulse. All patients, except one who underwent further treatment by vascular surgeons following closed reduction and fracture stabilisation, were managed by close observation and frequent serial neurovascular assessments for 48 hours per local institutional policy. Four patients eventually presented with neurological impairment; three exhibited median nerve impairment (posterolateral displacement), one had radial nerve symptoms (posteromedial displacement), and one experienced ulnar nerve palsy. The majority of cases were surgically addressed within 24 hours of injury, with an average operating time of 30-40 minutes. Finally, the average hospital stay for the treated patients was 2.5 days.

Carrying angle

The statistical analysis revealed that there was no significant difference (p = 0.713) between genders, pin configuration modes (p = 0.83), and time taken for treatment (p = 0.240). However, there was a correlation between Gartland’s grade and the preservation of the carrying angle (p = 0.43). Specifically, patients with Gartland Grade III were found to be at a higher risk of experiencing a loss of reduction.

Range of motion

There was no significant difference in the ROM based on the set parameters in the study. The p-values for sex, Gartland’s grade of fracture, pin configuration, and time to treatment were 0.760, 0.606, 0.333, and 0.333, respectively. This indicates that these factors did not significantly impact the ROM in the patients studied.

## Discussion

Supracondylar fractures are a frequent occurrence in paediatric elbow injuries. They typically occur during the first decade of life when growth is rapid, making it a crucial area of focus in paediatric traumatology. The management of these fractures presents a significant challenge due to several unresolved controversies. For instance, there is no clear consensus on the optimal pinning configuration that should be considered the gold standard, despite a growing trend among surgeons to prefer lateral entry fixation [12,13]. Additionally, the variables with the most significant impact on the prognosis of SCHFs are yet to be definitively established, which would enable clinicians to formulate a comprehensive treatment plan for their patients and provide informed consultations to both patients and parents.

Numerous authors have documented and suggested various factors that may influence the prognosis of SCHFs, leading to continued disagreement among experts. However, it is generally agreed that restoring the elbow’s ROM is the primary and desirable objective after this injury [14-17].

Surgeons widely use Gartland’s classification to assess the severity of SCHFs using radiographic criteria. The study by Barton et al. [11] demonstrated that this classification system has less intra and interobserver variability than any other system. However, it is essential to note that the classification alone cannot provide an algorithmic approach for determining conservative versus operative management. Factors such as displacement in the coronal and sagittal planes are critical in the decision-making process [16,17]. This classification system has evolved, with William et al. introducing the rotational fracture element and Leicht et al. adding the fourth-grade subset [18]. Despite the relevance of the newly introduced fourth subcategory, it is not yet widely used among clinicians. This could be because multidirectional instability can be assessed intraoperatively during manipulation and overzealous fracture reduction. This high-energy, unstable fracture pattern requires a unique surgical technique, stabilisation, and follow-up approach. The prognosis and natural history still need to be fully understood in the current literature [19,20]. Our patient cohort demonstrated that the high-grade Gartland variant IV had inferior functional recovery outcomes compared to the lower-grade counterparts. However, it is unclear if the long-term outcomes are improved [20]. Future dedicated studies are needed to answer this question.

This paper explicitly investigates the effects of various clinical parameters, including fracture pattern, time to surgery, mode of fixation, and age, on functional and aesthetic outcomes using Flynn’s criteria. Overall, the results were favourable, and no significant relationships were observed among the variables. A delay in surgery beyond 24 hours did not have any deleterious effects, as supported by multiple studies [5-9]. Although anecdotal reports have suggested that manipulation and reduction become more challenging with increasing delay in treatment, our cohort and other studies have not confirmed this claim.

As with any retrospective review research, our study has its expected limitations. Long-term patient follow-up examinations were only sometimes feasible, and multiple examiners of varying experience levels conducted them. While Flynn’s criteria assess the cosmetic and functional outcomes quite reliably and objectively, new patient outcome measures have emerged that might provide additional information and a new perspective.

The study has an adequate number of cases, which provides enough statistical power to make meaningful conclusions. It also explores the fourth subtype of Gartland’s classification, which is yet to be widely studied in the current literature.

## Conclusions

Generally, paediatric supracondylar fractures exhibit a favourable prognosis and typically lead to satisfactory functional outcomes wherein patients can resume their pre-injury activities without significant limitations. This may be attributable to the substantial potential for remodelling at a young age, particularly in those still experiencing growth. Moreover, positive outcomes may arise mainly from the anatomical and functional restorations and the affected region’s stabilisation.
